# Isolation and mass spectrometry analysis of urinary extraexosomal proteins

**DOI:** 10.1038/srep36331

**Published:** 2016-11-02

**Authors:** Siri Hildonen, Ellen Skarpen, Trine Grønhaug Halvorsen, Léon Reubsaet

**Affiliations:** 1 Department of Pharmaceutical Chemistry, School of Pharmacy, University of Oslo, Oslo, Norway; 2 Core facility for Advanced Light Microscopy, Department of Core Facilities, Institute for Cancer Research, Oslo University Hospital, Montebello, Oslo, Norway

## Abstract

The aim of the present study was to develop a LC-MS/MS-based proteomic analysis method of urinary exosomal proteins that has the potential to discover disease biomarkers. In short, urinary exosomes from healthy subjects were isolated by immunocapture on magnetic beads, detected by immunofluorescence and TEM, trypsin digested directly on the beads for an accelerated time with no addition of detergents before performing an LC-MS analysis of the trypsinate. To our knowledge, this is the first proteomic analysis of proteins displayed on the outer surface of exosomes. The outer exosome proteome may contain proteins that are of higher biomarker value compared to soluble cargo protein as the proteins projecting into the extracellular milieu might be more directly involved in physiological functions of exosomes. The proteomic analysis identified 49 proteins that were considered significant; the majority is involved in carbohydrate and lipid metabolism or in immune responses. Thirty of the proteins are linked to diseases. The developed proteomic method exploiting urinary exosomes might be of great value in search for diagnostic or prognostic biomarkers of especially metabolic and immune-related diseases.

Exosomes in cell cultures were first described in the 1980s by the groups of Stahl and Johnstone^1^,^2^,^3^. They observed that small vesicles, formed by inward budding of endosomes, were released into the extracellular space by fusion of the multivesicular body (MVB) with the plasma membrane. During the last three decades the interest of these 30–100 nm bilayered membrane vesicles has vastly increased after it was revealed that they are not merely cellular garbage cans. The findings that exosomes mediate intercellular communication^4^,^5^ sparked off functional studies on these small vesicles, secreted by cells into body fluid such as blood^6^, urine^7^, semen^8^, cerebrospinal fluid^9^, breast milk^10^ and saliva^11^. During 2015, there were close to 500 published papers in *pubmed*.*org* with exosomes in the title or abstract. Research has demonstrated that exosomes function in physiological as well as pathological processes^12^,^13^,^14^. Exosomes are mediators in immune responses^4^,^15^, metabolism^16^, angiogenesis^17^, apoptosis^18^,^19^, blood coagulation^19^,^20^ and are involved in renal^21^ and neurodegenerative diseases^22^,^23^,^24^ and development and progression of cancer^5^,^25^.

Currently there is a debate how to best isolate exosomes and how to differentiate them from other types of vesicles as well as quantifying these vesicles^26^. Differential centrifugation was the initial^2^ and is still the leading method to isolate exosomes. Besides differential centrifugation, filtration techniques and polymer precipitation are among other methods used to isolate exosomes^13^,^27^. A disadvantage of these techniques is that they do not isolate exosomes exclusively but also other types of vesicles, aggregates or cellular debris that have the same physical properties as exosomes^28^,^29^. In addition, the applied isolation technique is not always compatible with downstream LC-MS/MS analysis. Polymer precipitation of exosomes, for example, leads to contamination of the mass spectrometer resulting in repeating mass signals that interferes and even suppresses peptide ion peaks from the native sample. A chemical isolation method of exosomes based on immunocapture by antibodies against exosome-specific antigens has lately gained attention as it is considered a less laborious and more specific isolation and enrichment method of exosomes compared to the gold standard method of ultracentifugation^26^. However, it has not yet been established any exosome-specific marker or set of markers that are universal to exosomes excreted from all types of cells. Jørgensen *et al.* have done studies on this issue using antibody-based microarray technology to characterise and molecular profiling EV surface markers^30^,^31^. Researchers including exosomes in their studies are encouraged to follow the standardized isolation procedures proposed by the Society of Extracellular Vesicles^29^.

The preferred technique to detect exosomes is to directly observe them by electron microscopy (EM) as they fall below threshold of optical microscopy^4^. Indirect detection of exosomes is also performed by antibodies against exosome proteins applying techniques such as Western blotting or flow cytometry^29^. These techniques do not produce any visual proof of vesicles; they only confirm the presence of certain proteins that are considered specific for exosomes. It is important to note that the confidence of immune related methods is dependent on that the antibodies are specific against the antigens of interest.

Within the exosome research field the interest is not to merely isolate or detect exosomes but to characterise these vesicles regarding their composition and content and to reveal features that can be used in diagnosis, prognosis or therapy^14^,^32^,^33^. Exosomes are composed of membrane and cytosolic molecules including proteins, lipids and different types of RNA^34^. Identified mammalian exosome proteins, lipids and nucleic acids were listed in the Exocarta database^35^ which is now incorporated into a vesicle database Vesiclepedia (http://microvesicles.org)^36^. The database is based on data from principal researchers working on extracellular vesicles. Nearly 93 000 proteins are currently (October 2016) listed in Vesiclepedia. There are relatively few exosome characterisation studies based on LC-MS analysis; as few as 15 papers published during 2015 contain exosomes and mass spectrometry in their title or abstract when searching *pubmed*.*org*. Merely speculations, but the reasons might be that the main object has been to isolate to quantify exosomes and to state their origin. The research on exosomes has also had a high clinical focus and LC-MS/MS is not implemented yet as an instrument for routine analysis in the clinics. The lack of published work on LC/MS-based proteomic studies of exosomes might also be explained that there has been a reawake of genomics as these vesicles contain nucleotide acids in form of various types of RNA that carry genetic information between cells altering the cellular phenotypes^37^. Yet another reason might be that LC/MS analysis of exosomal membrane proteins or their soluble cargo protein is not a straightforward process. Gaining access to both protein fractions require aid of detergents that are not compatible with mass spectrometry analysis and their removal is necessary^38^.

Biomarker discovery studies are a huge field within proteomics. LC-MS/MS is to our opinion the method of choice for protein biomarker detection and identification. Unfortunately, mass spectrometry-based proteomics studies have been hampered by the complexity of body fluids^39^,^40^. Urine has become very attractive as a potential source of biomarkers alternative to blood plasma^41^. However, fractionation of urine is considered necessary as the number and dynamic range of proteins in normal human urine are beyond the capabilities of current MS-technology^42^,^43^. A recent study of human urine found that two third of the total proteins identified were not detected in unfractionated urine and that over half of the total identified proteins were associated with vesicles^43^. Urine is considered a rich reservoir of exosomes^7^,^44^ that are readily available by relatively inexpensively and noninvasive collection. Urine exosomes are exocytosed into urine by all renal epithelial cell types^45^. It is believed that the urinary exosome proteome may mirror disease specific changes not only perturbations due to renal diseases but also pathophysiology changes of other organs^44^.

The aim of this study was to develop a proteomic method to analyse the vesicular subfraction of urine that is directly compatible with LC-MS/MS. Digesting intact exosomes without detergent in the buffer were thought to give access to a subproteome of exosomes, the outer membrane associated proteins that might be of great biomarker value. Additionally, the methods applicability is tested on real samples.

To our knowledge this study is the first proteomic study of proteins protruding from the outer membrane layer of exosomes, recently there was published a paper based on a ultrafiltration and LC-MS/MS of the intraluminal proteome and peptidome of urinary extracellular vesicles^46^.

## Experimental procedures

### Materials

Vivaspin 20 MWCO 100 kDa (Sartorius, VS204) were used for concentrating urine for macromolecular structures. The following products from ThermoFisher Scientific (Waltham, Massachusetts, USA) were used for immunocapture and immunofluorescence: Dynabeads M-280 Tosylactivated, Dynabeads Protein G, Alexa Fluor 488 Donkey anti-Rabbit Antibody (A-21206), Alexa Fluor 594 Donkey anti-Mouse Antibody (A-21203), Alexa Fluor 647 Donkey anti-Goat Antibody (A-21447), Prolong Gold Antifade Mountant (P36934). Other products used for immunocapture were: anti-CD9 antibody (Abcam, ab65230), anti-CD63 antibody (Abcam, ab8219), anti-CD81 Antibody (Q-14) (Santa Cruz, sc-31234)

L-1-Tosylamide-2-phenylethyl chloromethyl ketone (TPCK) treated Trypsin, Trizma Base, Trizma HCl, formic acid (FA) and trifluoroacetic acid (TFA) were purchased from Sigma-Aldrich (St. Louis, MO, USA). All other chemicals used were of analytical grade.

### Urine collection, initial processing and storage

All experimental protocols were approved by University of Oslo and performed in accordance with relevant guidelines and regulations. Informed consent was obtained from all subjects in this study. Morning urine samples (100–150 mL) from healthy subjects were collected in sterile containers and centrifuged at 14000 × g fixed angle rotor (Eppendorf 5804, VWR International, Oslo) for 10 min at room temperature (within 1 hour of collection) to remove any particulate matter including cells and cell debris. The clarified urine was stored no longer than 24 hours at 4 °C before further analysis.

### Immunofluorescence confocal microscopy of whole-mounted exosome-bead complexes

Two μg of each antibody against native epitopes of CD8, CD63 and CD 81 were coupled to 100 μL Dynabeads M-280 Tosylactivated magnetic beads according to the manufacture’s manual (yielding three batches of 100 μL coated Dynabeads). To isolate exosomes from urine each bead solution was incubated with 20 mL diluted (50/50 with phosphate-buffered saline (PBS) buffer pH 7.4) clarified urine for 18 hours at 20 °C on a thermoshaker operating at 1000 rpm. Each bead solutions were washed with PBS 0.01% Tween20 pH 7.4 and solubilized in a total volume of 100 μL.

**Figure 1:**
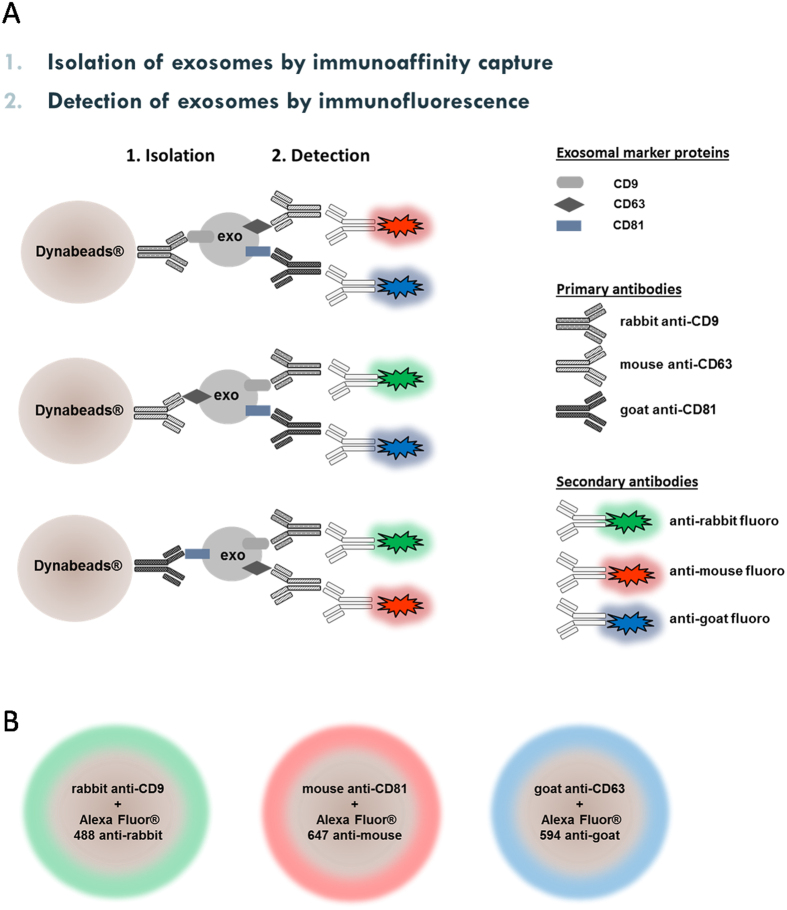
Illustrative overview of the exosome immunoisolation and immunodetection protocol.

Five μL of exosome-bead complexes were diluted to 50 μL by PBS-buffer pH 7.4 and incubated with the corresponding fluorescence secondary antibody according to the manufacture’s manual. Each 50 μL of bead solution was mounted on slides positioned on an in-house made device of circular magnets approximately 3 cm in diameter embedded in cardboard to help distributing the magnetic beads evenly over a small area (see supplementary, Fig. 1). To fixate and preserve the bead solutions on the slides 80 μL of an antifade mountant was applied on top of the bead solution before adding the cover plate and the slides were dried at room temperature overnight ahead of IF CM analysis.

The cells were examined with a Zeiss LSM780 confocal microscope (Carl Zeiss MicroImaging GmbH, Jena, Germany) equipped with an Ar-Laser Multiline (458/488/514 nm), a DPSS-561 10 (561 nm), a Laser diode 405-30 CW (405 nm), and a HeNe-laser (633 nm). The objective used was a Zeiss plan-Apochromat 63×/1.4 Oil DIC III. Image processing and visualization were performed with basic software ZEN 2010 (Carl Zeiss MicroImaging GmbH, Jena, Germany) and Photoshop CS4 (Adobe, Mountain View, CA).

### Transmission electron microscopy of whole-mounted exosome-bead complexes

Hundred and fifty mL clarified urine was ultrafiltrated and the urine was exchanged for PBS buffer 0.01% Tween pH 7.4 to an end volume of 5 mL using Vivaspin 20 MWCO 100 kDa centrifugal devices according to the manufacture’s manual. 125 μl of Dynabeads Protein G magnetic beads was added to the end volume of 5 mL and incubated overnight to remove any unspecific binding of particular matter or proteins to the beads. The supernatant was transferred to a new tube and the beads were wasted. Thirty μg antibodies against native epitopes on CD8, CD63 and CD 81 were coupled to 10 μL Dynabeads M-280 Tosylactivated magnetic beads or Dynabeads Protein G magnetic beads (diluted to 50 μL by PBS 0.01% Tween 20 pH 7.4) according to the manual. To isolate exosomes each bead solution was incubated 18 hours with 1 mL urine retentate (equivalent to 30 mL starting urine) at 20 °C on a thermoshaker operated at 1000 rpm. Each bead solutions was washed with PBS 0.01% Tween20 pH 7.4 and solubilized in a total volume of 100 μL.

Procedure for the bead eluate from tosylactivated beads: 10 μL of exosome eluate was fixed by 0.25% gluteraldehyde solution in 0.1 M sodium cacodylate on prewashed 100 mesh copper grids. The grid-mounted exosomes underwent negative staining with 1% uranyl acetate. After drying, the grids were examined and analysed by TEM (Philips CM100) operated at 80 kV. Images were recorded by an Olympus Quemesa Camera.

Procedure for the Dynabeads Protein G immunocapture experiments: 20 μL of exosome-bead solution were fixated by 1% glutaraldehyde and further by 1% osmium tetraoxide, negatively stained by 1% uranyl acetate. A microwave processor embedding protocol was applied: the fixed and stained exosome-bead solutions were dehydrated by increasing concentration of acetone at 250 watt 37 °C. Resin embedded by increasing amount of resin (EponTM Epoxy Resins, Miller Stephenson Chemical Company, CA, USA) and increasing temperature from 45 °C to 50 °C at 350 watt until 100% resin content was reached. Resin polymerisation was perfomed at 60 °C overnight. Selected areas were mounted on blocks. Ultra-thin sections (80 nm) were cut using a diamond knife at room temperature and sections were collected on 100 mesh copper grids and examined and analysed by TEM in a Philips CM100 microscope (North American Philips Co., Mahwah, NJ) operated at 80 kV. Images were recorded by an Olympus Quemesa Camera.

### Trypsin digestion of exosomes on beads

Eight μL of Dynabeads Protein G immunocaptured exosomes on beads (from TEM experiment) were tryptic digested (0.6 μg trypsin) in a total volume of 100 μL 50 mM Tris buffer pH 8 at 37 °C 1000 rpm for 30 minutes. Buffers and samples were heated to 37 °C prior to addition of trypsin to the samples. Supernatants of the tryptic digests were collected and the enzymatic activity was terminated by adding 100% formic acid to a final concentration of 2%.

### Tryptic digest salt clean-up

The tryptic digests were solid phase extraction (SPE) cleaned-up. Twohundred μL pipette tips were packed with 6 membrane stacks of C8 and 6 membrane stacks of C18 Empore Extraction Disks obtained from Phenomenex B.V. (Torrence, California). A centrifugal force of maximum 6000 × g was used to force buffer through the membrane stacks. The in-house SPE-tips were activated with 100 μL of 100% acetonitrile (MeCN) and equilibrated with 100 μL 0.1% FA. The tryptic digests were loaded onto the tips; bound peptides were washed with 100 μL of 0.1% FA and eluted by 100 μL of 80% MeCN. The eluate was evaporated to dryness and the peptides were resolubilized in 40 μL 3% MeCN, 0.01% TFA prior to the LC-MS/MS analysis.

Nano LC-MS/MS analysis Twenty μL of the proteolytic peptide mixtures were injected into the Chromeleon Xpress controlled Dionex HPLC system (Thermo Fischer, Bremen, Germany) and trapped on a C18 5 mm × 300 μm i.d. Acclaim PepMap 100 (5 μm) enrichment column (Dionex). The loading mobile phase, 20 mM FA and MeCN (98/2, v/v), was delivered at 10 μL/min for 4 minutes. The analytes were transferred to a 150 × 0.075 mm i.d. Acclaim PepMap 100 (pore size 100 Å, particle diameter 3 μm; Dionex) at 300 nL/min. The mobile phases consisted of A: 20 mM formic acid and MeCN (95/5, v/v) and B: 20 mM formic acid and MeCN (5/95, v/v). A linear gradient was run from 0% to 50% B in 60 minutes. Subsequently, the elution strength was increased to 100%. Total analysis time per run was 87 minutes. The LC setup was connected to an Xcalibur 2.0.7 controlled LTQ Discovery Orbitrap MS equipped with a Nano-ESI ion source (Thermo Fischer, Bremen, Germany). The nanospray ionization source was operated in the positive ionization mode (360 μm o.d. × 20 μm i.d. distal coated fused silica emitter, 10 μm i.d. tip (New Objective, Woburn, MA, USA)). The spray voltage was set at 2.2 kV. The heated capillary was kept at 150 °C. The capillary voltage was set at 45 V, and the tube lens was offset at 100 V. The mass spectrometer was operated in data-dependent positive ion-mode. Survey MS scans were performed in the orbitrap analyser at a resolution of 30 000 over a mass range between m/z 300–2000 Da with charge state disabled. Up to 6 most intense ions per scan were fragmented by collision induced dissociation (CID) at 35% relative collision energy, activation time of 30 ms, and analysed in the linear ion trap. The wide band activation option was enabled and dynamic exclusion of a time window of 15 seconds was used to minimize the extent of repeat sequencing of the peptides.

### Data interpretation

The MS raw files were processed with Proteome Discoverer 1.4 (Thermo Fischer, Bremen, Germany), using the Sequest algorithm, searching against reviewed human protein database generated from sequences obtained from Uniprot (May, 2015: 148764 entries). Enzyme specificity was set to trypsin. The initial parent and fragment ion maximum mass deviation was set to 20 ppm and 0.6 Da, respectively. The search included methionine oxidation as variable modification as well as N-terminal acetylation. Up to four missed cleavages were allowed and peptides had to be fully tryptic. A strict false discovery rate (FDR) was set to 0.01.

## Results and Discussion

### Isolation and detection of exosomes

Antibody coupled magnetic beads were used to isolate exosomes from urine. Considering that the antigen specificity of the antibodies used for isolation is high, the isolation methods will lead to purer exosome fractions compared to the gold standard method of isolating exosomes through ultracentrifugation. Antibodies against three tetraspanins, CD9, CD63 and CD81, were chosen to isolate exosomes. These three transmembrane proteins are involved in biogenesis of exosomes and believed to be ubiquitously expressed in exosomes^30^,^31^,^47^. However, it is important to note that the expression levels of each tetraspanins might vary considerably between individuals^30^.

To confirm the presence of exosomes and the specificity of the immunoextraction method, two different techniques were applied: IF EM and TEM.

### Detection of exosomes by IF EM

The anti-CD9, anti-CD63 and anti-CD81 antibodies used for immunocapture of exosomes were from three different species so that in theory the corresponding secondary antibody with a fluorochrome attached will bind specifically to only that of its matching specie. To confirm that an antibody had isolated exosomes one of the other two antibodies was used (once a time) in a second step (as outlined in Table 1 and Fig. 1a) for detection of the marker protein. This was thought to be an elegant way to confirm the presence of exosomal marker proteins without applying the more labor intense western blotting technique. As mentioned in the introduction, exosomes are too small to be observed in confocal microscopy but we expected to observe the magnetic beads, 2.8 μM in diameter, with fluorescence around the rim of the beads when the secondary antibody had bound to its corresponding primary antibody (Fig. 1b).

**Figure 2:**
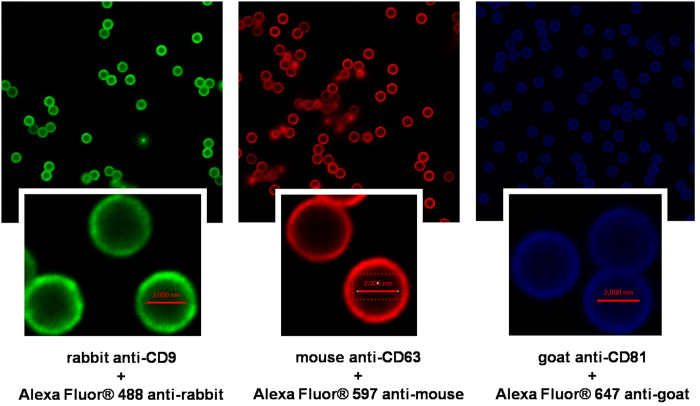
IF CM of the three antibody-secondary antibody combination that was used after immunocapturing exosomes on 2.8 μM magnetic beads.

Initially we did a dilution experiment to adjust the secondary antibody level to an amount that prevented fluorescence caused by non-specific binding of the antibody to the beads. The following negative controls were considered satisfactory: the beads alone, the beads incubated with urine, the beads incubated with any of the primary antibodies and urine and the beads incubated with urine and any of the secondary antibodies with fluorochrome attached. The beads displayed week intrinsic green fluorescence (data not shown), which was distinguished from the specific fluorescence from the fluorechrome by that the latter was only around the rim of the beads (i.e. they were bound to the outer surface). The autofluorescence from the beads was in contrast evenly distributed throughout the spherical object in image. This is explained by that the autofluorescence from the bead material will be a property throughout the beads whilst the fluorescence from the secondary antibodies will be only at the rim of the beads where the antibodies bind. We did observe fluorescence in all combination expected as schematized in Table 1 (Fig. 2) which confirms the presence of exosomal marker proteins around the rim of the beads. However, as mentioned this is not an observation of structures similar in size and structure to exosomes. When testing for cross reactivity we did observe that all of the three secondary antibodies displayed some reactivity against non-corresponding species but the signals were less intense than when the antibodies were combined with the matching primary antibody (data not shown).

### Detection of exosomes by TEM

**Figure 3:**
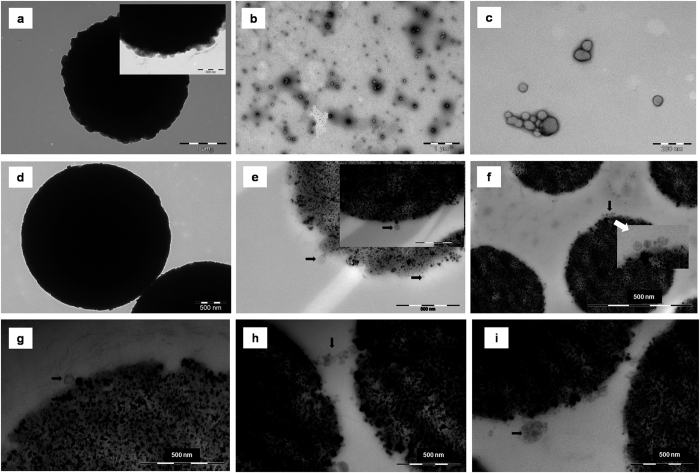
Transmission electron microscopy of clean and 2.8 μM magnetic beads. Arrows points to single vesicle when there are only few visible on the beads. Size bar displayed in the right lower corner of each image; (**a**) Clean Dynabeads M-280 Tosylactivated magnetic beads ; (**b**) Eluate solution from Dynabeads M-280 Tosylactivated magnetic beads used for CD63 immunocapturing in urine; (**c**) Eluate solution from Dynabeads M-280 Tosylactivated magnetic beads used for CD81 immunocapturing in urine; (**d**) Clean Dynabeads Protein G magnetic beads; (**e**) Dynabeads Protein G magnetic beads after CD9 immunocapturing in urine; (**f**) Dynabeads Protein G magnetic beads after CD63 immunocapturing in urine; (**g**) Dynabeads Protein G magnetic beads after CD81 immunocapturing in urine; (**h**) Dynabeads Protein G magnetic beads after CD63 immunocapturing. Arrow pointing to aggregates; (**i**) Dynabeads Protein G magnetic beads after CD63 immunocapturing. Arrow pointing to structure similar in morphology to MVBs.

To visually confirm the presence of exosomes bound to the beads and that the fluorescence from the beads corresponded with structures of morphology matching that of exosomes (bilayered, round-shaped of size 30–100 nM) we performed TEM of negatively stained exosomes. Unfortunately, the tosylactivated beads that we used for the IF CM experiments displayed an irregular surface making it impossible to observe any attached structures of small size due to the uneven surface (Fig. 3a). In other words, it was not possible to distinguish between clean beads and bead-exosome complexes. However, we did observe structures of exosomal size and morphology in an eluate solution: beads incubated with urine were treated with 1% formic acid for a few minutes and the eluate was dried to remove FA, resolubilised in PBS, mounted directly onto cupper mesh grids before analysed by TEM. Spherical structures were observed in the eluate from CD63-beads (Fig. 3b) and CD81-beads (Fig. 3c) but not in the CD9-beads eluate as the grid material disintegrated before an image were captured. For some unknown reason the grid material ruptured when the eluate was added and only after many attempts we successfully observed the structures from the eluate of CD81 and CD63 beads.

The isolation of exosome was repeated using the smoother protein G beads (Fig. 3d) in combination with a plastic embedding technique method to preserve the exosome-bead complexes. By applying this improved method, we did observe bilayered round-shaped structures attached to the beads when isolated by the CD9 (Fig. 3e), the CD63 (Fig. 3f) and the CD81 (Fig. 3g) antibodies. The sizes of the structures were determined to be fairly uniform from 30–100 nm consistent with studies of MVBs and exosomes from other tissues^7^. In contrast, TEM analysis of 100 kDa cutoff filtrate of urine revealed structures that were less uniform in size (Supplementary, Fig. 2). Filtration of urine will enrich any macrostructures present in the urine. Hardly quantitative, but we did observe less spherical structures on the CD9- and CD81 bound beads compared to the CD63 bound beads which displayed round shaped structures all around the surface of the beads (Fig. 3f). The few structures on the CD9 and CD81 bound beads might be due to many factors, discrepancies in the sample preparation at any step, less exosomes in the urine carrying these two markers, more weakly attached antibodies to the beads (the antibodies were not covalent bound to the beads) or weakly attached exosomes to the antibodies. Interestingly we did also observe aggregates made up of spherical structures on the CD63 beads (Fig. 3h), as well as structures similar in morphology of MVBs (Fig. 3i). The observation of aggregates might be explained by that a common protein in urine, uromodulin, forms network that leads to trapping of exosomes^48^. DTT treatment will disintegrate the network and release the exosomes^48^. We choose to leave out DTT treatment as the treatment probably would have negatively affected the immunocapture procedure in later steps. Uromodulin is also an inhibitor of trypsin so its presence should be avoided during trypsination. To get rid of the contaminating uromodulin we performed a centrifugation step as part of processing the urine samples, as outlined in the experimental procedures. However, we might not have removed all uromodulin-exosomes aggregates. To our knowledge there has not previously been reported MVBs in urine so we are not insisting that the image (Fig. 3i) displays a collection of vesicles enclosed by a membrane.

In conclusion, the TEM experiments confirmed the presence of structures corresponding to vesicles similar in size and morphology of exosomes on the surface of magnetic beads.

### Proteomic analysis of urinary vesicles by LC-MS/MS

#### Sample preparation considerations

LC-MS/MS analyses are hampered by laborious sample preparation ahead of the analysis. Included in this sample preparation, a time consuming digestion step is performed ahead of mass spectrometric analyses (bottom-up proteomics) as it is still not feasible to routinely analyse whole proteins (top-down proteomics). Our research group has shown that for a small mixture of proteins an accelerated digestion protocol (minutes instead of overnight digestion) performs better in terms of amino acid coverage of proteins, number of peptides generated, and peptide ion abundances than a conventional overnight digestion method^49^. Applying an accelerated digestion protocol of 30 minutes instead of the conventional overnight digestion method while exosomes are still on the beads will greatly reduce the sample preparation time of the exosome proteomic analysis protocol. By using a digestion buffer without detergents sample preparation time will further be reduced (as detergents have to be removed ahead of LC-MS/MS analysis). Additionally, absence of detergent will also lead to less sample complexity since it will enrich for a subset of the exosome proteome. Only peptides of proteins protruding from the outer membrane layer, such as transmembrane, covalent bound or membrane-associated proteins, will be released and collected for mass spectrometry analysis with no solubilizing detergents in the buffer. Cargo proteins of vesicles will be excluded. As the proteome of the outer membrane surface of exosomes is probably more directly involved in exosome functions than the proteins located internally of the vesicles it might be of more valuable for biomarker discovery. An example is aquaporin 2 which is an integral membrane protein of exosomes already exploited in clinical studies for 20 years^7^.

#### Protein hits after LC-MS/MS of trypsinate from bead-exosome complex solutions

The LC-MS/MS raw data were searched against a human database and forty-nine proteins, as listed in Table 2, out of 375 proteins were considered significant hits. Keratins were excluded as they are common contaminants in LC-MS/MS data. The mass spectrometry data were from triplicate analysis of trypsinate from immunocaptured exosomes using antibodies against the exosome marker proteins CD9,CD63 and CD 81 (the same three antibodies used in the IF CM and the EM experiments). To be considered significant protein hits three criteria had to be fulfilled: only proteins with two or more unique peptide hits identified were listed; they had to be present in the trypsinate from at least two of three parallel LC-MS/MS runs (for a few protein hits only presence in one parallel were allowed) for each antibody specific immunocapture experiment; lastly the coverage had to be higher compared to that of the negative controls (trypsinate from clean beads compared and trypsinate from clean beads incubated with urine). A table of all protein hits and their coverage can be found in Table 1, supplementary material. As seen from the supplementary table most protein hits display some coverage in negative controls, this might be due to carry-over effect that is not uncommon for mass spectrometric data. We ran two blank samples between each sample to minimize the effect. All significant 49 proteins were identified in all of the three antibody-specific immunocapture experiments (outlined in bold letters, Table 1, supplementary material). The high overlap between proteins detected by the three different antibodies confirms that the targeted antigens might be good overall markers of urinary exosomes. The data does not clearly point out any of the applied antibodies as being better than the others and the reason for observing less spherical structure on the CD9 and CD81 beads might be that the exosomes are lost during TEM sample preparation. We did also perform digestion of eluated exosomes (eluted off the beads with 1% formic acid). LC-MS/MS analysis of the eluate identified the same proteins but with no higher coverage, indeed in most cases lower coverage was observed compared to the trypsinate from nontreated exosome-bead complexes. Further trypsination of the beads after elution still did carry exosomes as the same proteins as in the untreated exosome-bead complexes were identified at approximately the same or lower coverage (data not shown). Applying higher trypsin amount in further experiments might lead to improved quality of the search data as there seems to be an excess of substrate compared to enzyme. We concluded that digesting directly on the beads performed better in overall. The data confirmed the presence of uromodulin, the protein seemed to stick to the LC column leading to a high carry-over effect as a rather high coverage was also present in the negative controls. It did not seem that any urinary protein bound unspecific to the beads as they would have been absent from MS-data of clean beads.

#### Coverage of identified proteins

Coverage of the 49 proteins in Table 2 ranged from 3% to 72%. Rather low coverage was expected for transmembrane proteins as trypsin has only access to the part of the protein protruding from the outer lipid bilayer. However also membrane bound or associated proteins were detected at rather low coverage. That the coverage within parallel runs varied more than we normally observe in LC-MS/MS can be explained by that the amount of each peptide in the sample are too low and hence vulnerable to be excluded as it will not reach the intensity threshold necessary for being selected for fragmentation. Low abundancy might be due to many factors, too few exosomes in the sample, too low amount of trypsin, digestion time, and inhibition by uromodulin or no optimal conditions for trypsin digestion, or due to posttranslational modifications.

#### Posttranslational modifications of identified proteins

As Table 2 reveals most of the proteins are phosphorylated, glycosylated or contain disulfide bridges (information collected from *uniprot*.*org*) neither of which we applied any technique to remove. Peptides containing these posttranslational modifications (PTMs) will not be detected when searching MS data against a search database if they are not taken into account. Manually comparing the peptide hit data with information of PTMs listed in *uniprot*.*org* we did confirm that whenever there was a cysteine involved in disulfide binding, or an O- or N-linked glycosylation site the peptide was not identified except for a few peptides containing N-linked glycosites that were detected by low confidence (data not shown). Performing reduction of disulfide bindings and deglycosylation of glycosites will most likely increase the coverage of identified proteins and probably add more proteins to the significant protein hit list. Our research group has recently demonstrated that N-linked deglycosylation leads to higher coverage of exosomal proteins (manuscript in preparation).

#### Localization, molecular function and biological function of identified proteins

*Uniprot*.*org* was exploited to get more information on each protein considered a significant hit such as localization, molecular function and biological function and association with diseases (Table 2). Most proteins were found to be localised to membranes, membrane bound or embedded in lipid bilayers. Further, by searching *vesiclepedia*.*org*, all proteins were found to be previously identified in urinary exosomes. When the protein was a transmembrane protein (nine in total) we did only identify peptides that was either listed as part of the luminal domain of lysosomes (two proteins, Table 2) or peptides within extracellular and not cytoplasmic domains (seven proteins, Table 2). The presence of peptides from luminal parts of lysosomal proteins is in accordance with the unique orientation of exosomes which mentioned are derived from invagination of lysosomal membranes forming MVBs^50^. No peptides of transmembrane domains were identified. To sum up, all 49 significant protein hits are of proteins already identified in exosomes and associated with membranes. Peptides from transmembrane proteins were mapped to part of the protein protruding from the outer bilayer of exosomes.

**Figure 4:**
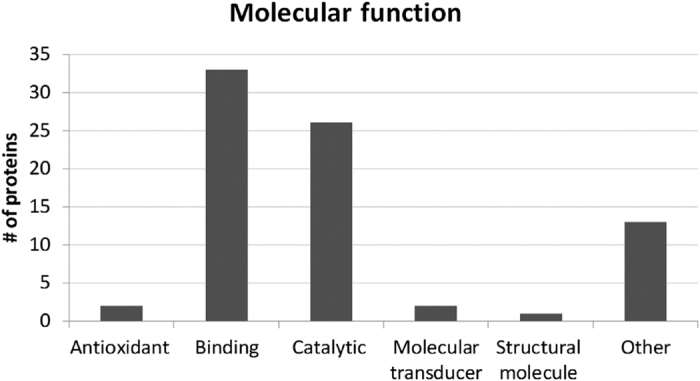
Molecular function based on gene ontology annotation of 49 proteins considered significant protein hits. The Software Tool for Researching Annotations of Proteins (STRAP) was used for generating GO ontology information and MS Excel was used for generating the chart. Each protein might be annotated to more than one category.

**Figure 5:**
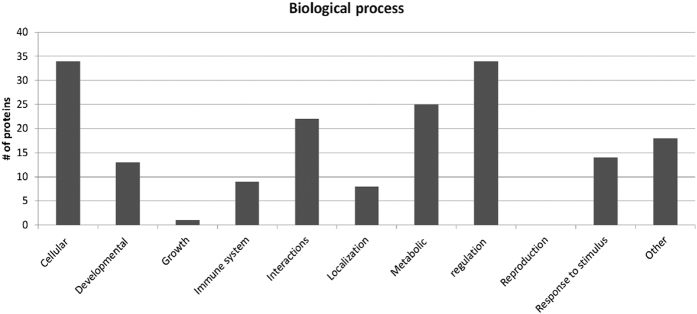
Biological function based on gene ontology annotation of 49 proteins considered significant protein hits. The Software Tool for Researching Annotations of Proteins (STRAP) was used for generating GO ontology information and MS Excel was used for generating the chart. Each protein might be annotated to more than one category.

To further investigate the protein hit data we used an open source software, The Software Tool for Researching Annotations of Proteins (STRAP) developed at the Cardiovascular Proteomics Center of Boston University School of Medicine (Boston, MA) (*www*.*bumc*.*bu*.*edu/cardiovascularproteomics/cpctools/strap/*) to generate a graphic illustration of molecular functions (Fig. 4) and biological functions (Fig. 5) based on gene ontology (GO). Note that a protein might have been given more than one function in the bar charts. STRAP automatically obtains GO terms associated with a protein list (based on accession numbers) using the freely accessible UniProtKB and EBI GOA databases^51^. Thirty-three of the 49 proteins considered as significant protein hits were annotated to be involved in binding, which is in accordance to the role of exosomes in communication and as players in the immune system. Our manually inspection of each protein though *uniprot*.*org* confirmed that at least 10 proteins are assigned to have functions of the immune system (Table 2). Interestingly there was recently published a paper comparing the urinary, non-urinary and urinary exosome proteomes based on GO annotation. The conclusion of the study was that metabolic proteins are particularly represented in urinary exosomes compared to exosome from other body fluids or compared to the proteome of total urine^52^. The result of this GO-study is in accordance to our findings: 26 proteins were given catalytic molecular functions (Fig. 4) and 25 proteins were found to be involved in metabolic processes (Fig. 5). Further, by manually interpretation of each protein through *uniprot*.*org*, 22 of the 49 proteins considered as significant protein hits, were found to be involved in carbohydrate (Table 2: 16 proteins) or lipid metabolism (Table 2: 6 proteins). The biological functions annotated to the 49 proteins apart from metabolic or immune related were cellular, developmental, interactions, localization, regulation and response to stimulus with none related to reproduction. These findings suggest that proteins of the outer part of urinary exosomes are involved in many of the diverse biological functions assigned to exosomes. Our data further suggest that the outer proteome of urinary exosomes probably do not have any major roles in reproduction.

#### Identified proteins linkage to diseases

Thirty of the 49 proteins in our study are linked to various diseases (Table 2). Since their linkage to diseases might not be directly related to their appearance in exosomes and as this is not a comparative proteomic study of diseased and non-diseased states we decided not to look further into any of the diseases. However, a certain disease, lysosomal storage disease (LSD) is worth mentioning as it is overrepresented in the data: 12 of the 30 proteins linked to diseases, are proteins that when aberrant can lead to LSD (Table 2). LSD is a rare inherited metabolic disorder that results from defects in lysosomal function mostly as a result of nonfunctioning or dysfunctional lysosomal metabolic enzymes^53^. Ten proteins found in our study linked to LSD are enzymes localized to the lumen of lysosomes (Table 2). That these proteins are localised to the outer surface of exosomes in our study is again in accordance with the shift in orientation of proteins bound to membranes when lysosomes develop into MVBs as mentioned earlier. That enzymes that normally exhibit narrow pH ranges functioning inside the acidic lysosomes are found on the outer surface of urinary exosomes in much less acidic fluid is more convincing explained by that these metabolic enzymes are being wasted out of the organism bound to exosomes. This is contrast to the theory suggested by Bruchi *et al.* that the biological function of metabolic enzymes enriched in urinary exosome is that aerobic energy are important for the lifetime of exosomes and/or their functioning as metabolic effectors^52^.

#### Identified proteins as potential biomarkers

Even if 30 of the proteins in Table 2 are involved in diseases and by that considered as potential biomarkers, it does not mean that they can be easily implemented into diagnosis of diseases. Diagnosis that is based on measuring quantitative changes between normal and diseased stages of the proteome has to be validated which is not an easy task of urinary samples. There is no consensus within the field of how to best normalise human urine that displays huge inter- and intra-individual variations in volume and protein content^29^,^54^. However, the potential of discover potential biomarkers in urine has previously been demonstrated by our group. Four of the proteins in this proteomic study of exosomes were earlier suggested by our group as biomarkers of renal rejection events of kidney transplant patients after performing a proteomic study of urine depleted for the most abundant proteins^55^. This previous study identified eleven proteins, functioning either in immune or growth regulated responses, that were significantly upregulated in early stages of acute renal rejection ahead of increased creatinine levels (which is a nonspecific marker of acute rejection). Anyway, it might turn out that the biggest potential for implementing discovered urinary biomarkers into diagnosis of diseases is not based on quantitative changes of proteins or peptides but rather based on single exosomal proteins that appears upon disease. An example of an exosomal protein that is successfully been implemented in early diagnosis of pancreatic cancer based merely on its appearance is Glypican-1^56^.

#### Concluding remarks

Exosomes are small vesicles of endocytic origin secreted into most body fluids. Research on exosomes has intensified after they were found to be involved in both normal physiological processes as well as in pathophysiological conditions. We have developed a urinary exosome isolation protocol based on magnetic bead immunocapture on magnetic beads that requires less expensive equipment and less expertise and leads to purer exosome fractions than the most widely used isolation method ultracentrifugation. The immunocapture method using three different antibodies against known antigens of exosomes confirmed vesicular structures bound to the beads. To be less time-consuming the tryspination time was greatly reduced compared to the conventional overnight digestion and performed directly on the beads with no addition of detergents. With no lipid dissolving concentration of detergent added, only the parts of proteins protruding from the outer membrane bilayer of exosomes will be trypsinated. This study is to our knowledge the first proteomic study of extraexosomal urinary proteins. We identified 49 proteins that were considered significant protein hits, all previously identified in urinary exosomes and all were found to be associated or bound to membranes. When applicable, the proteins were confirmed to have an orientation that is consistent with the swap from the inner to the outer membranes when exosomes develop from lysosomes.

The GO annotation of the 49 identified proteins confirmed that exosomes contain proteins involved in diverse biological functions. Although no attempt was made in this study to identify changes in the human urinary proteome that can be related to pathophysiology, several proteins involved in disease were identified including a high incidence of proteins related to metabolic disorders. Especially identifying biomarkers of lysosomal storage disease seems promising as 12 identified proteins are related to LSD. Additionally, several proteins identified in this study have previously been suggested as biomarkers of early stage of renal rejection in kidney transplant patients. Applying the developed protocol to comparative studies of urine from healthy subjects versus subjects of metabolic or renal diseases will hopefully lead to discovery of extraexosomal biomarkers that can be used for diagnosis and/or prognosis.

## Additional Information

**How to cite this article**: Hildonen, S. *et al.* Isolation and mass spectrometry analysis of urinary extraexosomal proteins. *Sci. Rep.* **6**, 36331; doi: 10.1038/srep36331 (2016).

**Publisher’s note:** Springer Nature remains neutral with regard to jurisdictional claims in published maps and institutional affiliations.

## Supplementary Material

Supplementary Information

Supplementary Table 1

## Figures and Tables

**Table 1:** Schematic overview of the exosome immunoisolation and immunodetection protocol.

1. Isolation step					2. IF CM detection step				
								theoretical result	experimental result
Dynabeads®	+	anti-CD9 Ab (rabbit)	+	urine sample	anti-CD63 Ab (mouse)	+	Alexa Fluor® 594 anti-mouse Ab	+	+
					anti-CD81 Ab (goat)	+	Alexa Fluor® 647 anti-goat Ab	+	+
					−	+	Alexa Fluor® 488 anti-rabbit Ab	+	+
Dynabeads®	+	anti-CD63 Ab (mouse)	+	urine sample	anti-CD9 Ab (rabbit)	+	Alexa Fluor® 488 anti-rabbit Ab	+	+
					anti-CD81 Ab (goat)	+	Alexa Fluor® 647 anti-goat Ab	+	+
					−	+	Alexa Fluor® 594 anti-mouse Ab	+	+
Dynabeads®	+	anti-CD81 Ab (goat)	+	urine sample	anti-CD9 Ab (rabbit)	+	Alexa Fluor® 488 anti-rabbit Ab	+	+
					anti-CD63 Ab (mouse)	+	Alexa Fluor® 594 anti-mouse Ab	+	+
					−	+	Alexa Fluor® 647 anti-goat Ab	+	+
Dynabeads®	+	no Ab	+	urine sample	−	+	Alexa Fluor® 594 anti-mouse Ab	−	−
					−	+	Alexa Fluor® 647 anti-goat Ab	−	−
					−	+	Alexa Fluor® 488 anti-rabbit Ab	−	−

**Table 2:** Table of proteins considered significant hits.

	Gene name	Accession	Name	Coverage range %	PTMs	Location	Peptide hits domains	Molecular function	Biological Function	Disease associated	Vesiclepedia/urine
GLUCOSE METABOLISM16 proteinswhen no gene symbol is found, gene number it is similar to is denoted by*	GAA	P10253	Lysosomal alpha-glucosidase	23–39	S, N-linked G	lysosome (lumen and membrane associated)	na	glucosidase	degradation of glygogen to glucose in lysosomes	Pompe disease, Glycogen storage disease type II (lysosomal storage disease)	Y/Y
	GAA*	B7Z5V6	cDNA FLJ57046, highly similar to Lysosomal alpha-glucosidase	17–30	S, N-linked G	lysosome (lumen and membrane associated)	na	glucosidase	carbohydrate metabolisme	Pompe disease, Glycogen storage disease type II (lysosomal storage disease)	Y/Y
	CTSA	P10619-2	Lysosomal protective protein	5–10	S, N-linked G	lysosome (lumen)	na	carboxypeptidase	carbohydrate metabolisme	Galactosialidosis (lysosomal storage disease)	Y/Y
	HYAL1	Q12794-2	Isoform 2 of Hyaluronidase-1	6–16	S, N-linked G	lysosome (lumen)	na	glycosidase	carbohydrate metabolisme	Mucopolysaccharidosis 9 (lysosomal storage disease)	Y/Y
	GLB1	P16278	Beta-galactosidase	6–17	N-linked G	cytoplasma, lysosome (lumen)	na	hydrolase	carbohydrate metabolisme	GM1-gangliosidosis 1,2 and 3, Mucopolysaccharidosis 4B (lysosomal storage diseases)	Y/Y
	NAGLU	P54802	Alpha-N-acetylglucosaminidase	10–23	N-linked G	lysosome (lumen)	na	glycosidase	carbohydrate metabolisme	Mucopolysaccharidosis 3B (lysosomal storage disease)	Y/Y
	NEU1	Q99519	Sialidase-1	3–16	N-linked G	cell membrane, cytoplasmic vesicle, lysosome (lumen and membrane associated)	na	glycosidase	carbohydrate and lipid metabolisme	Sialidosis (lysosomal storage disease)	Y/Y
	PGAM2	P15259	Phosphoglycerate mutase 2	4–26		cytosol, exosome (extracellular)	na	hydrolase	glucose metabolisme	Glycogen storage disease 10 (lysosomal storage disease)	Y/Y
	PGLS	O95336	6-phosphogluconolactonase	11–33	P	cytoplasma	na	hydrolase	carbohydrate metabolisme	Diabetic nephropathy	Y/Y
	ALDOB	P05062	Fructose-bisphosphate aldolase B	13–27	P	cytoplasma	na	lyase	glycolysis	Hereditary fructose intolerance	Y/Y
	DCXR	Q7Z4W1	L-xylulose reductase	5–21	P	peripherial membrane	na	reductase	carbohydrate metabolisme	Pentosuria	Y/Y
	B4GALT1	P15291-2	Isoform short of Beta-1,4-galactosyltransferase 1	5–18	S, N-linked G	SP TM: Golgi, plasma membrane.	Luminal	transferase	glycolysis	Congenital disorder of glycosylation 2D	Y/Y
	GAPDH	P04406	Glyceraldehyde-3-phosphate dehydrogenase	10–25	P	cytoplasma, cytoskeleton, membrane	na	oxidoreductase, transferase	glycolysis, apoptosis		Y/Y
	MAN1A1	P33908	Mannosyl-oligosaccharide 1,2-alpha-mannosidase IA	19 -46	S, N-linked G	SP TM: membrane (exosome, ER, Golgi)	Luminal	glycosidase	carbohydrate metabolisme		Y/Y
	CPN2	P22792	Carboxypeptidase N subunit 2	12–32	N-linked G	secreted extracellulr space (exosomes)	na	enzyme regulator	carbohydrate metabolisme. Immune		Y/Y
	GSTA1	P08263	Glutathione S-transferase A1	6–18		cytoplasma, exosomes	na	transferase	metabolisme		Y/Y
**LIPID METABOLISM6 proteins**when no gene symbol is found, gene number it is similar to is denoted by*	ASAH1	B1B5Q3	N-acylsphingosine amidohydrolase 1	11–36	N-linked G	lysosome (lumen), exosomes	na	sphingosine hydrolase	lipid metabolisme	Farber lipogranulomatosis (lysosomal storage disease)	Y/Y
	ASAH1*	B3KUZ6	cDNA FLJ40980 fis, clone UTERU2014464, highly similar to ACID CERAMIDASE	19–41	N-linked G	lysosome (lumen), exosomes	na	sphingosine hydrolase	lipid metabolisme	Farber lipogranulomatosis (lysosomal storage disease)	Y/Y
	GM2A	P17900	Ganglioside GM2 activator	6–10	S, N-linked G	lysosome (lumen), exosome (extracellular), plasma membrane (cytoplasmic)	na	hydrolase	lipid metabolisme	GM2-gangliosidosis AB (lysosomal storage disease)	Y/Y
	APOE	P02649	Apolipoprotein E	4–18	P, O-linked G	ubiquitous (membrane associated)	na	binding	lipid, sterol and cholesterol metabolisme and transport	Lipoprotein glomerulopathy; Alzheimer disease, Amyloidosis, Hyperlipidemia	Y/Y
	CEL	P19835	Bile salt-activated lipase	11–33	S, N- and O-linked G	secreted extracellular space	na	hydrolase	lipid metabolisme	Maturity-onset diabetes of the young 8 with exocrine dysfunction	Y/Y
	LCAT	P04180	Phosphatidylcholine-sterol acyltransferase	12–23	S, N- and O -linked G	secreted extracelluar space	na	acyl transferase	cholesterol, lipid, sterol, steroid metabolisme	Lecithin-cholesterol acyltransferase deficiency, Fish-eye disease	Y/Y
**IMMUNE RESPONSES10 proteins**	CTSD	P07339	Cathepsin D	11–3 0	S, N- and O-linked G	lysosome (lumen), secreted extraqcellular space	na	lysosomale endoprotease	autophagy, metabolic degradation, immune	Ceroid lipofuscinosis, neuronal, 10 (lysosomal storage disease), Alzheimer disease	Y/Y
	C3	P01024	Complement C3	3–16	S, N-Linked G	extracellular space, exosomes	na	promotes binding	immune	Hemolytic uremic syndrome atypical 5, Complement component 3 deficiency, Macular degeneration age-related 9;	Y/Y
	C4B	P0C0L5	Complement C4-B	5–12	S, N-linked G	secreted extracellular space, exosomes	na	binding	immune	Systemic lupus erythematosus	Y/Y
	KNG1	P01042-2	Kininogen-1	13–29	P, S, N-linked G	secreted extracellular space, exosomes	na	protease inhibitor	immune, blood coagulation	High molecular weight kininogen deficiency	Y/Y
	SERPING1	P05155	Plasma protease C1 inhibitor	27–41	S, N- and O-linked G	secreted extracellular space	na	protease inhibitor	immune; blood coagulation	Hereditary angioedema	Y/Y
	TPP1	O14773	Tripeptidyl-peptidase 1	9–33	S, N-linked G	lysosome (lumen), exosomes	na	endopeptidase	many, immune	Ceroid lipofuscinosis, neuronal 2, Spinocerebellar ataxia autosomal recessive 7	Y/Y
	OLFM4	Q6UX06	Olfactomedin-4	13–29	S, N-linked G	secreted extracellular space	na	binding	cell adhesion, immiune	Pancreatic cancer?	Y/Y
	PGLYRP2	Q96PD5	N-acetylmuramoyl-L-alanine amidase	12–27	P, N-linked G	secreted (membrane associated)	na	hydrolase	immune		Y/Y
	CLU	E7ERK6	Clusterin	25–36	S, N-linked G, P	ubiquitous including vesicles	na	chaperone	many (Immune, apoptosis)		Y/Y
	CLU	P10909-2	Isoform 2 of Clusterin	29–32	P, S, N-linked G	ubiquitous including vesicles	na	chaperone	many (Immune, apoptosis)		Y/Y
**OTHER17 proteins**when no gene symbol is found, gene number it is similar to is denoted by*	SERPINA1	P01009	Alpha-1-antitrypsin	9–46	P, N-linked G	extracellular (ER, exosomes)	na	peptidase inhibitor	acute phase, blood coagulation	Alpha-1-antitrypsin deficiency	Y/Y
	ALB	P02768	Serum albumin	3–12	P, S, N-linked G	ubiquitous (exosomes)	na	binding	transport	Hyperthyroxinemia, Analbuminemia	Y/Y
	NID1	P14543-2	Isoform 2 of Nidogen-1	10–23	N-linked G	secreted extracellular space	na	binding	cell adhesion	Bethlem myopathy, Ullrich congenital muscular dystrophy	Y/Y
	ACTB	P60709	Actin, cytoplasmic 1	25–51		cytoplasma	na	binding	ubiquitous	Dystonia juvenile-onset, Baraitser-Winter syndrome 1	Y/Y
	EFEMP1	Q12805-4	EGF-containing fibulin-like extracellular matrix protein 1	10–35	S, N-linked G	secreted extracellular space	na	growth factor	ubiquitous	Doyne honeycomb retinal dystrophy	Y/Y
	EGF	P01133-3	Isoform 3 of Pro-epidermal growth factor	21–37	S, N-linked G	SP TM (PM and vesicles)	extracellular	growth factor	ubiquitous		Y/Y
	LMAN2*	A8K7T4	cDNA FLJ75774, highly similar to Homo sapiens Vesicular integral-membrane protein VIP36 (LMAN2), mRNA	20–41	na	membrane	na	carbohydrate binding	carbohydrate binding		Y/Y
	QSOX1	O00391	Sulphydryl oxidase 1	9–19	S, N-linked G	SP TM membrane	extracellular	sulhydryl oxidase	redox homeostasis, regulator macroautophagy		Y/Y
	PIGR	P01833	Polymeric immunoglobulin receptor	5–14	P, S, N-linked G	SP TM, membrane (exosome)	extracellular	receptor	transcytose in epithelial cells		Y/Y
	AHSG	P02765	Alpha-2-HS-glycoprotein	3–17	P, S, N- and O-linked G	secreted extracellular space	na	inhibitor	promotes endocytosis		Y/Y
	SERPINA5	P05154	Plasma serine protease inhibitor	44–72	N-and O-linked G	ubiquitous	na	protease inhibitor	lipid transport		Y/Y
	PROZ	P22891	Vitamin K-dependent protein Z	9–23	S, N and O-linked G	secreted extracellular space	na	protease	blood coagulation		Y/Y
	ITIH4	Q14624-2	Inter-alpha-trypsin inhibitor heavy chain H4	10–29	N- and O-linked G	secreted extracellular space	na	protease inhibitor	acute phase		Y/Y
	VASN	Q6EMK4	Vasorin	15–24	S, N-linked G	SP TM (lysosomal, exosome, PM)	extracellular	TGF-beta binding	hypoxia, redox		Y/Y
	LRRC15	Q8TF66	Leucine-rich repeat-containing protein 15	9–21	N -linked G	membrane	extracellular	binding	cell migration		Y/Y
	ZG16B	Q96DA0	Zymogen granule protein 16 homolog B	12–49	N -linked G	secretetd extracellular space	na	carbohydrate binding	retina homeostasis		Y/Y
	PCDHGC3	Q9UN70-2	Isoform 2 of protocadherin gamma-C3	5–17	N-linked G	SP TM membrane (exosomes, PM)	extracellular	binding	cell adhesion		Y/Y

Information retrieved from own data, *uniprot*.*org* and *microvesicles*.*org*. Table displays gene name, accession number, name, coverage range in three parallel MS-runs, known PTMs, known location, peptide hit domain in identified protein, known molecular function, known biological function, disease association and if previously identified in exosome samples and in urine denoted with Y (yes).
