# Supplementary material for: Isolation and mass spectrometry analysis of urinary extraexosomal proteins
Source: Sci Rep. 2016 Nov 2;6:36331. doi: 10.1038/srep36331 (PMC5090863; doi:10.1038/srep36331)
Supplement: Supplementary Information [file srep36331-s1.pdf]

Isolation and mass spectrometry analysis of urinary extraexosomal proteins

Siri Hildonen, Ellen Skarpen, Trine Grønhaug Halvorsen, Léon Reubsaet

Figure 1

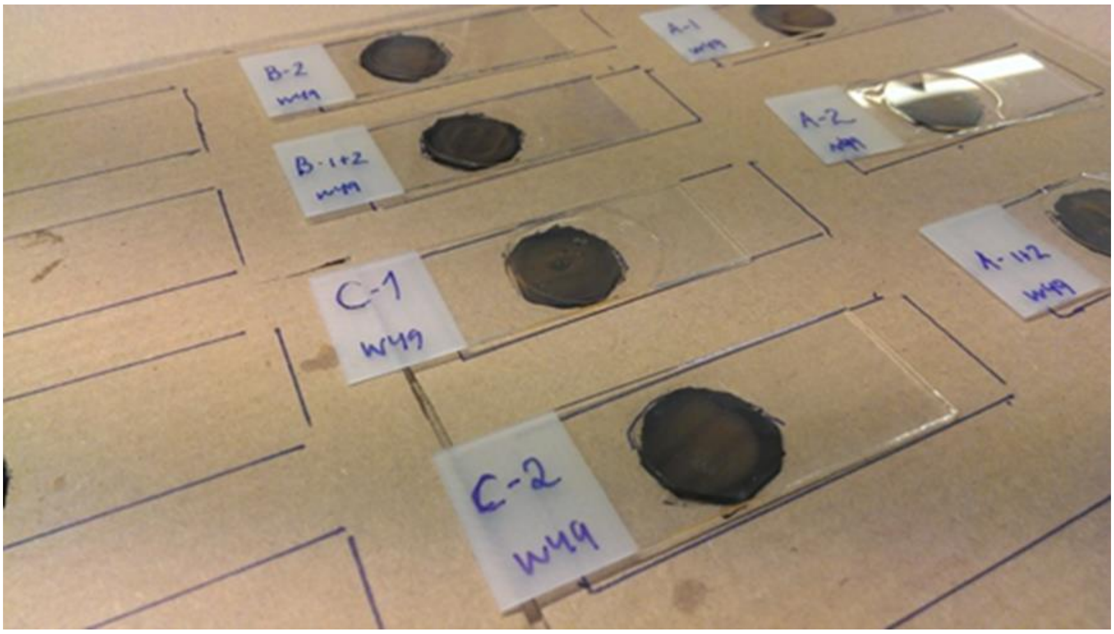

Figure 2

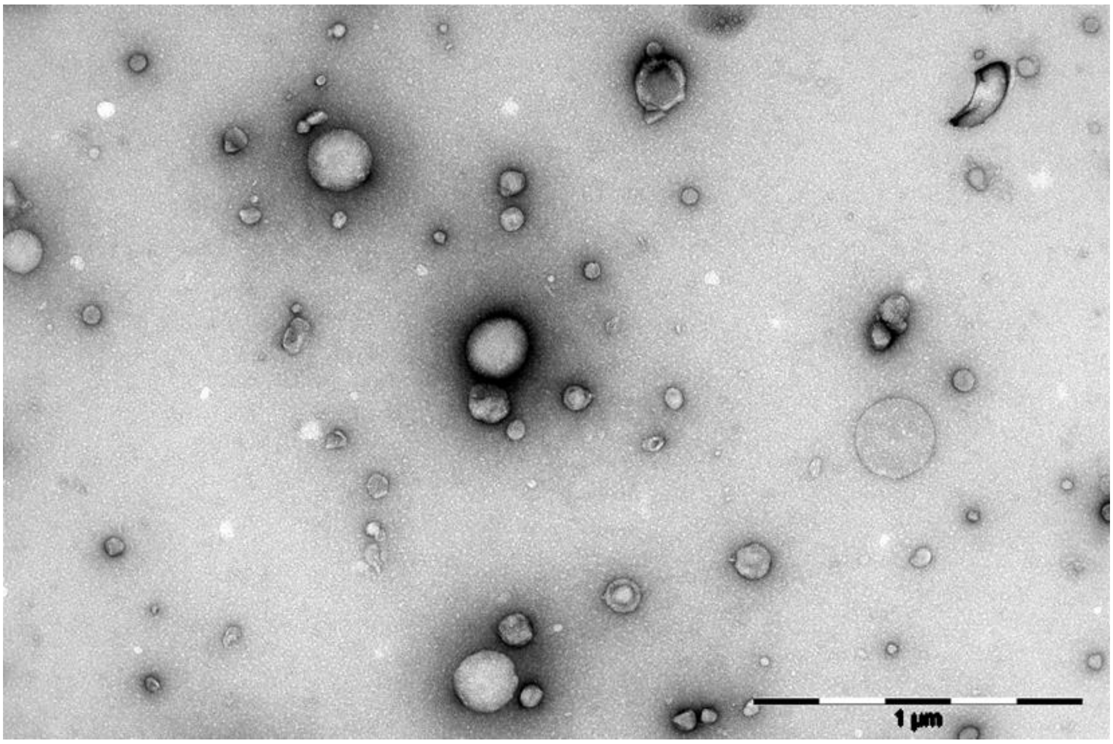

Supplementary legends

Figure 1. The in-house device setup with magnets embedded in cardboard for mounting exosome-bead complexes onto microcope slides.

Figure 2. Transmission electron microscopy of of 100 kDa cutoff filtrate of urine

Table 1. Table of all protein hits and their coverage after searching LC-MS/MS raw data against a human database
